# Comparative effects of traditional Chinese and Western migraine medicines in an animal model of nociceptive trigeminovascular activation

**DOI:** 10.1177/0333102417728245

**Published:** 2017-08-24

**Authors:** Yonglie Zhao, Margarida Martins-Oliveira, Simon Akerman, Peter J Goadsby

**Affiliations:** 1 Department of Neurology, University of California, San Francisco, CA, USA; 2 Basic and Clinical Neurosciences, Institute of Psychiatry, Psychology and Neuroscience, King’s College London, London, UK

**Keywords:** Gastrodin, ligustrazine, migraine, headache, trigeminocervical complex

## Abstract

**Background:**

Migraine is a highly prevalent and disabling disorder of the brain with limited therapeutic options, particularly for preventive treatment. There is a need to identify novel targets and test their potential efficacy in relevant preclinical migraine models. Traditional Chinese medicines have been used for millennia and may offer avenues for exploration.

**Methods:**

We evaluated two traditional Chinese medicines, gastrodin and ligustrazine, and compared them to two Western approaches with propranolol and levetiracetam, one effective and one ineffective, in an established *in vivo* rodent model of nociceptive durovascular trigeminal activation.

**Results:**

Intravenous gastrodin (30 and 100 mg/kg) significantly inhibited nociceptive dural-evoked neuronal firing in the trigeminocervical complex. Ligustrazine (10 mg/kg) and propranolol (3 mg/kg) also significantly inhibited dural-evoked trigeminocervical complex responses, although the timing of responses of ligustrazine does not match its pharmacokinetic profile. Levetiracetam had no effects on trigeminovascular responses.

**Conclusion:**

Our data suggest gastrodin has potential as an anti-migraine treatment, whereas ligustrazine seems less promising. Interestingly, in line with clinical trial data, propranolol was effective and levetiracetam not. Exploration of the mechanisms and modelling effects of Chinese traditional therapies offers novel route for drug discovery in migraine.

## Introduction

Migraine is a common disorder of the brain (1,2), recognized by the World Health Organization as the sixth most common cause of disability globally, and the most common neurologic cause of disability (3). The pain arises from activation, or the perception of activation, of nociceptive trigeminal durovascular afferents (4,5). Based on human work demonstrating that dural afferent activation leads to the perception of pain (6–8), experimental model systems have been developed to explore the physiology and pharmacology of the trigeminovascular nociceptive pathways as a means of developing new treatments (9,10). Despite a promising development pipeline (11), current therapies leave much room for improvement, particularly for migraine prevention (12).

**Figure 1.**
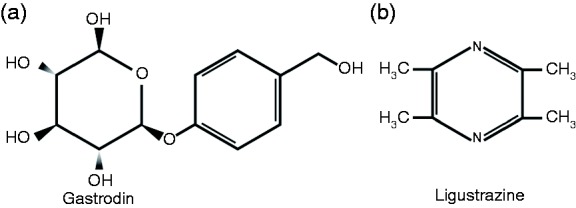
Chemical structure of (a) gastrodin and (b) ligustrazine.

The English-language scientific literature in headache remains dominated by European and American studies and therapeutic approaches. As the Far East emerges, approaches now less familiar and used for millennia may offer new opportunities and insights. Tian ma (Rhizoma Gastrodiae, Figure 1(a)) and Chuanxiong (Rhizoma Chuanxiong, Figure 1(b)) are two traditional Chinese medicines that are both widely used for treating primary headaches, as well as other neurological disorders, in China (13,14). There is currently limited clinical or preclinical data validating their potential therapeutic efficacy in headache, particularly written in English.

Our aim was to test the efficacy of these two traditional Chinese medicines used for the treatment of primary headache in an established *in vivo* rodent model of trigeminovascular dural nociception. We compared the effects of these medicines to an established migraine preventive, propranolol (12), and levetiracetam, whose published placebo-controlled clinical failed its primary endpoint (15).

## Methods

### Surgical preparation

All experiments were conducted under license of the UCSF Institutional Animal Care and Use Committee, and conforming to the National Institute of Health Guide for the Care and Use of Laboratory Animals, and adhered to the guidelines of the Committee for Research and Ethical Issues of IASP (16).

The surgical preparation and recording methods have been reported in detail previously (17,18). Fifty four male Sprague-Dawley rats (250–370 g) were anesthetized using a single dose of sodium pentobarbital (60 mg kg^−1^ i.p.; Nembutal, Diamondback Drugs, Scottsdale, AZ) for induction, and propofol (20–25 mg kg^−1^ h^−1^ i.v., Propoflo, Abbott, Abbott Park, IL, USA) for maintenance throughout the experiment. The left femoral artery and vein were cannulated for continuous blood pressure recording and administration of the anesthetic. The right femoral vein was cannulated for drug administration. Temperature was maintained at 37℃ ± 0.5℃ using a homeothermic blanket system with a rectal probe. Rats were placed in a stereotaxic frame and ventilated with oxygen-enriched air, 3 to 5 ml/min, 75 to 90 strokes per minute. End-tidal CO_2_ was monitored and kept between 3.5% and 4.5%. Blood pressure, temperature and end-tidal CO_2_ data were displayed and saved throughout the entire experiment on a personal computer using an online data analysis system (Power 1401plus and Spike 5.2 CED, UK). A sufficient depth of anesthesia was judged by the absence of paw withdrawal and corneal blink reflex. At the end of each experiment animals were euthanized with intravenous administration of a lethal dose of pentobarbital and phenytoin sodium (Euthasol).

### Stimulation of the middle meningeal artery and recording in the TCC

**Figure 2.**
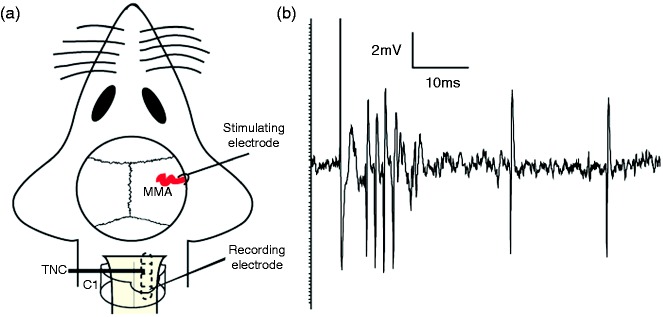
(a) The basic experimental setup with dural stimulation and recording in TCC and (b) an original tracing from a typical unit responding to dural stimulation with latencies in the Aδ-fiber and C-fiber range.

The skull was exposed and access to the dura mater and middle meningeal artery (MMA) was created by drilling away part of the left parietal bone, leaving the dura mater intact. The area was covered in mineral oil to protect from dehydration. For extracellular recordings of trigeminocervical complex (TCC) neurons, the muscles of the dorsal neck were separated, a C1 laminectomy was performed and the dura and pia mater were carefully removed using a dissection microscope and fine forceps at the level of the caudal medulla. A tungsten microelectrode, with a recording impedance of 1.0 to 2.2 MΩ (measured at 1 kHz in 0.9% saline), was lowered into the TCC near the dorsal root entry zone (Figure 2(a)). For the location of the optimal recording site, the electrode was advanced or retracted in 5 µm steps using a piezoelectric motor-driven microelectrode positioner attached to a micromanipulator (Kopf 202111L, USA). The electrical signal obtained from the recording electrode was filtered (bandwidth approximately 300 Hz–20 KHz and 60 Hz noise eliminator) and amplified (total gain 20 K–30 K), and was fed to a gated amplitude discriminator and an analog-to-digital converter (Power 1401, CED, UK) connected to a notebook computer for collection, analysis, and storage (Spike 5.2, CED, UK). Additionally, the signal was fed to a loudspeaker through a power amplifier (Neurolog NL120, Digitimer, UK) for audio monitoring and displayed on an oscilloscope to assist the isolation of unit activity from background noise.

A bipolar stimulating electrode was then placed onto the dura mater above the middle meningeal artery and connected to a stimulus isolation unit (SIU5; Grass Instruments, Quincy, MA). Primary trigeminal afferents were stimulated supramaximally with square wave pulses generated by a Grass S88 stimulator (10–20 V, 0.1–0.3 ms, 0.5 Hz). Neurons were further characterized by responses to noxious pinch and innocuous brush of the cutaneous facial receptive field in the ophthalmic dermatome and were classified as wide dynamic range. Responses were analyzed using post-stimulus histograms with a sweep length of 100 ms and a bin width of 1 ms that separated Aδ-fiber (approximately 3–20 ms) and C-fiber (20–80 ms) firing, based on conduction velocities of primary afferents (19,20) and the distance of the dural meninges to the TCC, via the trigeminal ganglion (18). Baseline responses consisted of trains of 20 stimuli delivered to the dura mater at five minute intervals. Background activity was analyzed as cumulative rate histograms, in which activity gated through the amplitude discriminator was collected into successive bins.

### Drug administration

After obtaining reliable baselines to dural electrical stimulation, calculated as the mean of four stimulation series, either gastrodin (30 and 100 mg/kg, Kunming Pharmaceutical CORP, China), levetiracetam (10 mg/kg, Sigma-Aldrich, USA), ligustrazine (3 and 10 mg/kg, Rongsheng Pharmaceutical CORP, China), or propranolol (3 mg/kg, Tocris Cookson Ltd, USA) were administered intravenously over two minutes, with normal saline as the vehicle. Post-stimulus histograms were repeated for up to two hours after drug intervention every 10 minutes.

### Statistical analysis

The data collected as post-stimulus histograms after electrical stimulation of the dura mater for Aδ-fibers represent the number of cells fired over at least a 10 ms period in the region 5–20 ms and for C-fibers 20–80 ms, post-stimulation over the 20 collections. Spontaneous activity was measured in cell firings per second (Hz). All data are expressed as mean ± SEM. Statistical analysis was performed using an ANOVA for repeated measures with Bonferroni *post-hoc* correction for multiple comparisons used to measure the time course of significant drug intervention, using a 95% confidence interval. If Mauchly’s test of sphericity was violated, we made appropriate corrections to degrees of freedom according to Greenhouse–Geisser. Student’s paired *t*-test for *post-hoc* analysis was used to test for the time points of significance, using the average of the four baselines for comparison, again using the criteria of Bonferroni correction. Statistical significance was set at *p* < 0.05 (SPSS 18.0)

## Results

In all experiments temperature and end-tidal CO_2_ were kept at physiological levels.

### Electrophysiology

Recordings were made from 54 neurons (in 54 rats) responsive to dural stimulation. Neurons were located in the dorsal horn of the trigeminocervical complex, which includes the cervical C1 region of the spinal cord and its transition to the trigeminal nucleus caudalis, at a range of depth of 100–860 µm. The evoked neuronal firing unit responded to dural stimulation with latencies in the Aδ-fiber and C-fiber range (Figure 2(b)). Intravenous administration of 0.9% sodium chloride had no effect on ongoing spontaneous firing (*F*_6,42_ = 0.90, *p* = 0.54, *n* = 8) or stimulus-evoked Aδ-fiber responses (*F*_1.1,7.7_ = 1.1, *p* = 0.34, *n* = 8). There was no effect of any treatment on C fiber responses; the data are not further reported.

### Blood pressure effect

**Figure 3.**
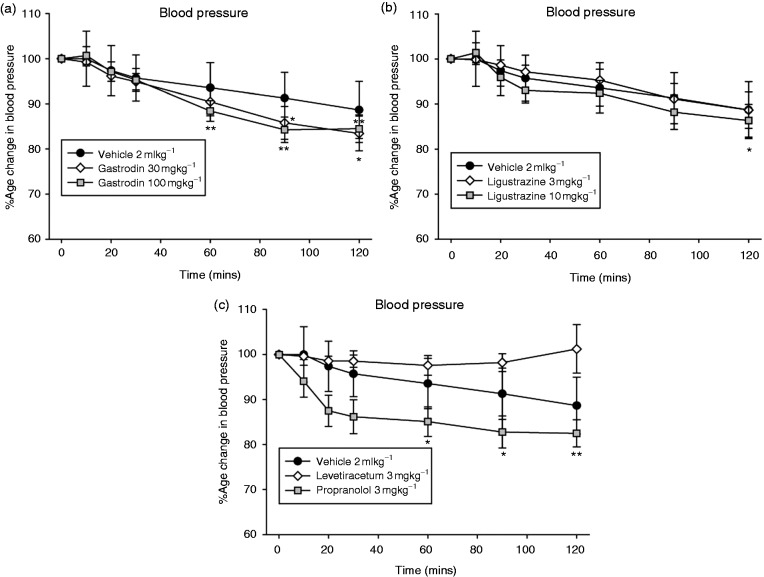
Effect of migraine preventives on mean arterial blood pressure. (a) Intravenous administration of gastrodin led to a long lasting dose-dependent decrease of blood pressure that reached significance after 75–90 min (30 mg/kg) and 45–60 min (100 mg/kg). (b) Intravenous ligustrazine also led to a dose-dependent decrease in blood pressure that reached significance after 105–120 min (10 mg/kg). (c) Intravenous propranolol led to a long lasting decrease in blood pressure that reached significance after 45–60 min. Neither intravenous saline (vehicle) nor levetiracetam had any effect on blood pressure. Data are represented as mean ± SEM. *p < 0.05, **p < 0.01, compared with an average of the four baselines, using Student’s paired t test.

Intravenous administration of saline did not significantly affect blood pressure (*F*_1.5,10.7_ = 2.5, *p* = 0.14, *n* = 8). Intravenous administration of gastrodin led to a significant decrease in blood pressure (30 mg/kg; *F*_1.3,8.5_ = 15.5, *p* = 0.003, *n* = 8; 100 mg/kg; *F*_2.3,16.0_ = 15.1, *p* = 0.0001, *n* = 8, Figure 3(a)) compared with baseline. Intravenous administration of gastrodin (30 mg/kg) caused a maximum decrease of blood pressure of 17 ± 4% (t_7_ = 4.3, *p* < 0.05) at 105–120 min, and similarly with 100 mg/kg by 16 ± 3% (t_7_ = 5.5, *p* < 0.01), at 75–90 min when compared with baseline. Likewise, intravenous administration of ligustrazine significantly decreased blood pressure (3 mg/kg; *F*_1.2,8.4_ = 6.3, *p* = 0.031, *n* = 8; 10 mg/kg; *F*_2.0,13.8_ = 8.9, *p* = 0.003, *n* = 8, Figure 3(b)). Intravenous administration of ligustrazine (3 mgkg^−1^) caused a maximum decrease of blood pressure of 11 ± 4% (t_7_ = 2.7, *p* < 0.05) at 105–120 min, and similarly with 10 mg/kg by 14 ± 4% (t_7_ = 3.7, *p* < 0.01), at 105–120 min when compared with baseline. Intravenous administration of levetiracetam (10 mg/kg) did not cause any significant change in blood pressure (*F*_1.3,7.7_ = 0.24, *p* = 0.72, *n* = 7, Figure 3(c)). Intravenous administration of propranolol (3 mg/kg) caused a significant decrease in blood pressure (*F*_6,36_ = 18.5, *p* = 0.0001, *n* = 7, Figure 3(c)) maximally by 18 ± 3% compared with baseline at 105–120 min.

### Effects of traditional Chinese preventives on nociceptive trigeminovascular neurotransmission

#### Gastrodin

**Figure 4.**
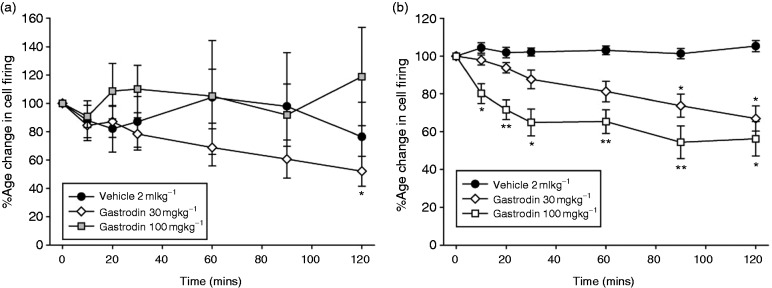
Effects of gastrodin on nociceptive trigeminovascular activation. Time course changes in (a) ongoing spontaneous trigeminal neuronal firing and (b) the average response of intracranial dural-evoked Aδ-fiber trigeminal neurons to intravenous administration of saline and gastrodin (30 and 100 mg/kg). Only gastrodin (30 mg/kg) caused inhibition of ongoing spontaneous trigeminal neuronal firing, after 105–120 min. Gastrodin caused a dose-dependent inhibition of intracranial dural evoked Aδ-fiber trigeminal firing, after 75–90 min for 30 mg/kg and 5–10 min after 100 mg/kg. Saline had no effect on trigeminovascular neuronal firing. Data are represented as mean ± SEM. *p < 0.05, **p < 0.01 compared with an average of the four baselines, using Student’s paired t test.

Intravenous administration of gastrodin (30 mg/kg) significantly inhibited ongoing spontaneous activity in the TCC (*F*_1.8,12.6_ = 5.0, *p* = 0.029, *n* = 8) (Figure 4(c)) maximally by 52 ± 11% (*t*_7_ = 4.5, *p* < 0.05) after 105–120 min compared to baseline, whereas the higher dose did not have any effect (100 mg/kg, *F*_1.8,12.8_ = 0.54, *p* = 0.58, *n* = 8). Intravenous administration of gastrodin (30 and 100 mg/kg) caused a dose-related inhibition of stimulus-evoked Aδ-fiber responses in the TCC (30 mg/kg; *F*_6,42_ = 12.5, *p* = 0.0001, *n* = 8; 100 mg/kg; *F*_6,42_ = 10.14, *p* = 0.0001 *n* = 8; Figure 4(b)). There was a maximum inhibition of stimulus-evoked Aδ-fiber activity of 33 ± 7% at 105–120 min after 30 mg/kg gastrodin (t_7_ = 4.7, *p* = 0.002), and 46 ± 9% at 75–90 min after 100 mg/kg gastrodin (t_7_ = 4.8, *p* = 0.002; Figure 4(b)), compared to baseline.

#### Ligustrazine

**Figure 5.**
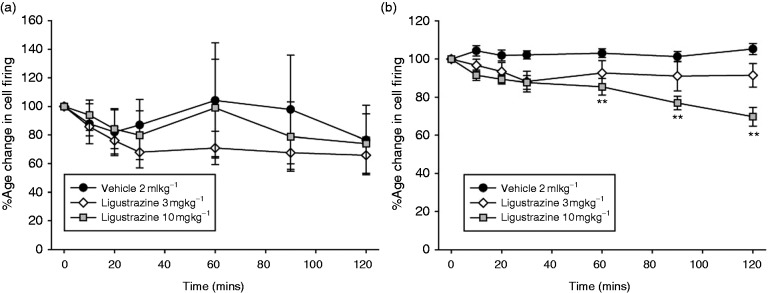
Effects of ligustrazine on nociceptive trigeminovascular activation. Time course changes in (a) ongoing spontaneous trigeminal neuronal firing and (b) the average response of intracranial dural-evoked Aδ-fiber trigeminal neurons to intravenous administration of saline and ligustrazine (3 and 10 mg/kg). Ligustrazine had no effect on ongoing spontaneous trigeminal neuronal firing, but caused a dose-dependent inhibition of intracranial dural-evoked Aδ-fiber trigeminal firing. Only ligustrazine (10 mg/kg) caused inhibition of intracranial dural-evoked Aδ-fiber trigeminal firing, after 75–90 min. Data are represented as mean ± SEM **p < 0.01 compared with an average of the four baselines, using Student’s paired t test.

Intravenous administration of ligustrazine had no significant effect on ongoing spontaneous activity in the TCC (3 mg/kg; *F*_1.2,8.1_ = 2.36, *p* = 0.163, *n* = 8, 10 mg/kg; *F*_1.6,10.8_ = 2.5, *p* = 0.13, *n* = 8; Figure 5(a)). Intravenous administration of ligustrazine (3 and 10 mg/kg) caused an inhibition of stimulus-evoked Aδ-fiber responses in the TCC (3 mg/kg; *F*_6,42_ = 1.51, *p* = 0.20, *n* = 8, 10 mg/kg; *F*_2.4,16.8_ = 10.7, *p* = 0.001, *n* = 8; Figure 5(b)), maximally after 105–120 min by 30 ± 5 % (*t*_7_ = 6.2, *p* = 0.0001) compared to baseline.

### Effects of Western migraine preventives on nociceptive trigeminovascular neurotransmission

**Figure 6.**
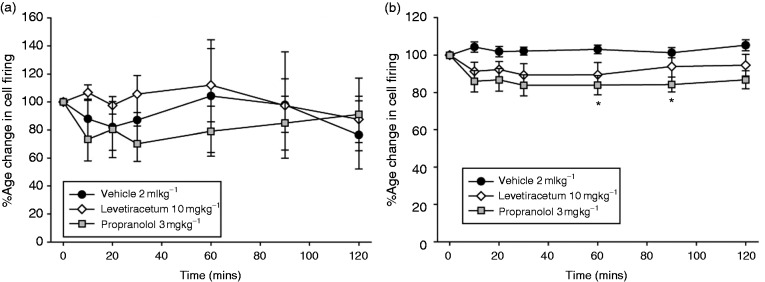
Effects of propranolol and levetiracetam on nociceptive trigeminovascular activation. Time course changes in (a) ongoing spontaneous trigeminal neuronal firing and (b) the average response of intracranial dural-evoked Aδ-fiber trigeminal neurons to intravenous administration of saline, propranolol (3 mg/kg) and levetiracetam (10 mg/kg). Levetiracetam had no effect on either ongoing spontaneous or intracranial dural-evoked Aδ-fiber trigeminal neuronal firing. Propranolol had no effect on ongoing spontaneous trigeminal neuronal firing, but caused significant inhibition of intracranial dural-evoked Aδ-fiber trigeminal firing after 75–90 min. Data are represented as mean ± SEM. *p < 0.05 compared with an average of the four baselines, using Student’s paired t test.

Intravenous administration of levetiracetam, at an approximate clinical dose (10 mg/kg), had no effect on stimulus-evoked Aδ-fiber activity (*F*_6,36_ = 0.90, *p* = 0.51, *n* = 7) or spontaneous activity (*F*_1.2,7.4_ = 0.65, *p* = 0.48, *n* = 7) in the TCC (Figure 6(a)–(b)). Intravenous administration of propranolol (3 mg/kg) significantly reduced stimulus-evoked Aδ-fiber activity (*F*_6,36_ = 2.5, *p* = 0.039, *n* = 7), maximally after 75–90 min by 16 ± 4% (*t*_6_ = 3.9, *p* = 0.008), compared with baseline. Propranolol had no effect on ongoing spontaneous TCC activity (*F*_1.9,11.6_ = 0.10, *p* = 0.397, *n* = 7; Figure 6(a)–(b)).

## Discussion

In this study, we demonstrated in an *in vivo* model of trigeminovascular nociceptive activation that the traditional Chinese medicines gastrodin and ligustrazine were both able to inhibit nociceptive dural-evoked neuronal firing in the trigeminocervical complex. Interestingly, only a low dose of gastrodin affected ongoing spontaneous firing. As a comparison, we also used the established migraine preventive propranolol (12) and the ineffective compound levetiracetam; propranolol significantly inhibited dural-evoked responses, and levetiracetam did not. These data support investigation of the use of gastrodin and ligustrazine in the treatment of migraine. Moreover, the data illustrate the utility of exploring traditional Chinese medicine approaches as signposts to development of new migraine medicines.

Gastrodin (p-hydroxymethylphenyl-β-D-glucopyranoside) is one of the main bioactive components in Tian ma (*Gastrodia elata*), which is claimed to have sedative, anticonvulsive and neuroprotective effects. While there is no clear evidence in the English-language literature of controlled trials for the use of gastrodin in the treatment of migraine, anecdotal evidence in the traditional Chinese medicine literature indicates that it is used to treat various neurological disorders. In China, it is approved for the treatment of primary headaches including migraine, and other central nervous system diseases including vertigo, insomnia, neuralgia, neurasthenia, and epilepsy (13). Previous preclinical studies have indicated gastrodin may be effective in pain disorders. It can attenuate nociceptive behavioral responses in streptozotocin-induced diabetic neuropathy, and abolishes abnormal hyperexcitability of dorsal root ganglion neurons caused by this diabetic neuropathy, perhaps by normalizing transient sodium currents and potassium currents (21).

Gastrodin was able to inhibit nociceptive dural-evoked neuronal firing in the trigeminocervical complex in a dose-related fashion, and also affected ongoing spontaneous firing at its lowest dose. Gastrodin caused a dose-dependent reduction in mean arterial blood pressure. In this context, the inhibitory effects of gastrodin on dural-evoked neurons started after five minutes with the highest dose, whereas blood pressure was only reduced after 60 minutes. We note that treatments not altering trigeminocervical responses, notably saline vehicle, also had this blood pressure sagging effect over time. Taking the time course, modest effect and parallel effect with vehicle, we consider the blood pressure an unlikely explanation for the trigeminal inhibitory effect of gastrodin. However, we cannot exclude the possibility, and could explore this in the future by using microiontophoresis, which would test the local effect on trigeminocervical neurons without any issue of systemic changes. Moreover, we cannot exclude a possibly non-receptor mediated effect of gastrodin on blood pressure. From a migraine perspective, it is known that calcitonin gene-related peptide (CGRP) is released during migraine (22,23) and can trigger migraine when given exogenously (24). In cultured rat trigeminal ganglion cells, gastrodin caused a dose-dependent reduction of CGRP- mRNA expression, as measured using immunoreactivity, that was similar to flunarizine (25). Gastrodin also reduced phosphorylated extracellular signal-regulated kinase 1/2 protein (ERK1/2), which might imply that the intracellular ERK1/2 signaling pathway is involved in gastrodin-inhibited CGRP up-regulation (25). It is thought that dural-evoked neuronal firing in the trigeminocervical complex is mediated in some way by the release of CGRP centrally where either pre- (26,27) or post-synaptic mechanisms (28) are possible. Previous studies have shown that gastrodin is not markedly brain penetrant. In Sprague Dawley rats, gastrodin (50 mg/kg, intravenous) had a CSF/plasma ratio of only 5.5% (29), reaching a maximum CSF concentration after 10 minutes of approximately 3.75 µg/mg and half-life of approximately 35 minutes, although there was still detectable levels after two hours. The maximum concentration in plasma is almost immediate with a half-life of approximately 25 minutes. In our study, we used 100 mg/kg and observed significant inhibition after 10 minutes, similar to the pharmacokinetic studies, which lasted for at least two hours, and may be accounted for by the larger dose. It is not known how much gastrodin in the brain is necessary to exert a significant physiological effect, but the µg/ml concentration is similar to the ligustrazine data below (30), which is considered highly brain penetrant. Our data suggest that gastrodin may exert its effects by either preventing the release of CGRP from pre-synaptic central projections or inhibiting the post-synaptic effects of the ERK1/2 downstream signaling pathway to inhibit the firing of second-order trigeminal nociceptive neurons.

Ligustrazine (2,3,5,6-tetramethylpyrazine) is an active alkaloid originally isolated from the Chinese herb Ligusticum chuanxiong Hort. It is considered to have anti-inflammatory, anti-oxidant and anti-fibrotic effects. In the traditional Chinese medicine literature, it is widely reported as being used for the treatment of cerebrovascular and cardiovascular diseases, such as cerebral ischemia, coronary heart disease, hypertension and pulmonary hypertension, as well as pain disorders, including headache (31,32). Tetramethylpyrazine has both anti-inflammatory and analgesic effects in various established pain models, and these effects are thought to be mediated by actions at the P2X3 receptor on primary afferent neurons (33–35). Here, ligustrazine had no effect on ongoing spontaneous neuronal firing and only inhibited nociceptive dural-evoked neuronal firing in the trigeminocervical complex at the highest dose (10 mg/kg) after one hour. Importantly, it only affected blood pressure at the highest dose after two hours. Previous studies have demonstrated that ligustrazine in Sprague Dawley rats (10 mg/kg, intravenous) has a T_max_ of approximately five minutes in CSF and the cerebral cortex and nearly 15 minutes in blood plasma, and a half-life of approximately 35–40 min in all tissue types sampled (30), with a CSF/plasma ratio of nearly 50 % and cortex/plasma ratio of nearly 20 %. These data imply ligustrazine has good and rapid access to the brain, and the delayed effect observed may reflect a metabolite.

Beta-blockers, including propranolol, are established migraine preventives used clinically, although their mechanism of action is unclear. Chronic administration of propranolol at 20 mg/kg per day in rats is able to reduce the frequency of cortical spreading depression, which is considered to be the experimental correlate of migraine aura (36). It has also been shown that propranolol inhibits dural nociceptive trigeminothalamic neurons, when applied directly into ventroposteromedial thalamic (VPM) nucleus using microiontophoresis (37), suggesting a potential central locus of action. Our data demonstrate that propranolol (3 mg/kg) had no effect on ongoing spontaneous firing, but effectively inhibited nociceptive dural-evoked neuronal firing in the trigeminocervical complex after 60 minutes. The data act as a useful positive control.

Levetiracetam is a pyrrolidone derivative with a distinctive anti-epileptic pharmacologic profile. It is thought to exert its effects on the synaptic vesicle protein (SV2A) brain binding site, which differs from the targets of other anti-epileptic drugs (38). The migraine data are conflicting. One study of chronic daily headache failed on its primary endpoint (15), while another in migraine has been reported to be positive against placebo (39). Our data suggest this medicine would not be effective, and this is in line with the author’s (PJG) clinical experience. If unpublished data exists, its public availability would help better understand this question.

In summary, our data demonstrate effects of two traditional Chinese medicine treatments of migraine: gastrodin and ligustrazine. The agents performed well against propranolol and better than levetiracetam, so that we see a possible role for these agents at least in migraine prevention. The model of dural nociceptive trigeminovascular activation is likely to activate a pathway that predicts migraine effects not limited to acute or preventive strategies, and recent data with calcitonin gene-related peptide (CGRP) antagonists demonstrates anti-migraine compounds can have both effects (40,41). Western medicine has much to learn from traditional Chinese approaches and exploring mechanisms of treatments from that tradition, and dissecting out the role of the components of traditional Chinese therapies may yield insights to progress in the development of new treatments for migraine.

## Article highlights

- Practitioners using traditional Chinese medicines report that they can be extremely useful for migraine prevention.
- Two traditional Chinese medicine treatments, gastrodin and ligustrazine, inhibited dural-evoked trigeminocervical neuronal activity in an experimental model of the nociceptive pathway of migraine.
- The established Western migraine preventive, propranolol, was also effective in the same experimental model as gastrodin and ligustrazine, while levetiracetam, which has no positive placebo controlled data in migraine, was not.
- Traditional Chinese medicine offers a largely untapped opportunity to develop therapies and explore mechanisms that will likely aid migraine drug discovery.
